# A System for Neuromotor Based Rehabilitation on a Passive Robotic Aid

**DOI:** 10.3390/s21093130

**Published:** 2021-04-30

**Authors:** Marco Righi, Massimo Magrini, Cristina Dolciotti, Davide Moroni

**Affiliations:** 1 National Research Council of Italy, ISTI Area della Ricerca CNR, Via G. Moruzzi 1, 56124 Pisa, Italy; massimo.magrini@isti.cnr.it (M.M.); davide.moroni@isti.cnr.it (D.M.); 2 Computer Science Department, University of Pisa, Lungarno Pacinotti, 56126 Pisa, Italy; 3 Department of Traslational Research and New Technologies in Medicine and Surgery, University of Pisa, Lungarno Pacinotti, 43, 56126 Pisa, Italy; c.dolciotti@gmail.com

**Keywords:** computer graphics, motion analysis, prognostics and health, rehabilitation robotics, robotics

## Abstract

In the aging world population, the occurrence of neuromotor deficits arising from stroke and other medical conditions is expected to grow, demanding the design of new and more effective approaches to rehabilitation. In this paper, we show how the combination of robotic technologies with progress in exergaming methodologies may lead to the creation of new rehabilitation protocols favoring motor re-learning. To this end, we introduce the Track-Hold system for neuromotor rehabilitation based on a passive robotic arm and integrated software. A special configuration of weights on the robotic arm fully balances the weight of the patients’ arm, allowing them to perform a purely neurological task, overcoming the muscular effort of similar free-hand exercises. A set of adaptive and configurable exercises are proposed to patients through a large display and a graphical user interface. Common everyday tasks are also proposed for patients to learn again the associated actions in a persistent way, thus improving life independence. A data analysis module was also designed to monitor progress and compute indices of post-stroke neurological damage and Parkinsonian-type disorders. The system was tested in the lab and in a pilot project involving five patients in the post-stroke chronic stage with partial paralysis of the right upper limb, showing encouraging preliminary results.

## 1. Introduction

### 1.1. Current Epidemiological Data on Stroke Incidence and and Post-Stroke Outcome

The decay of the motor capacity is a relatively common condition in the elderly population [1] and can inhibit the skills necessary to perform daily life activities. Aging is not the only cause of this functional decline and consequent loss of the aged’s abilities [2]. The World Health Organization (WHO) reports that more than 15 million people in the world are affected by cardiovascular disease, and among them, a non-negligible percentage, 10–20% of cases, have experienced cerebrovascular disease or stroke. The cerebral stroke is in 85% of cases of the ischemic type and in 15% of cases of the hemorrhagic type, in this case affecting younger subjects (usually hypertensive or carriers of vascular malformation) [3]. Stroke remains the second cause of mortality and the first of disability [3,4,5]. In 2019, the Italian National Health Service reported that there were 20,000 cases of stroke in Italy, 80% of which were first episodes, while 20% were recurrences [6]. In the coming years, the forecast is that there will be a growing need for specific medical expertise, physiotherapy, and support from caregivers to better assist patients [6].

In terms of outcome for the survivors (about 85%), the consequences of the stroke are very variable. The effects of the stroke can be represented by a speech and language disorder (dysarthria and aphasia) or by *motor deficits*, *temporary* or *permanent* (plegia and paresis, respectively), which may be more or less severe and extensive [3]. Two therapeutic modalities exist for post-stroke neurorehabilitation of the upper limbs: task-oriented therapy and virtual reality [7]. The first method is based on the administration of intrinsic and extrinsic feedback during a defined protocol of exercises, while the second concerns the use of a virtual environment, in which it is possible to set the frequency and mode of administration of feedback during therapy [7,8]. Feedback can be of intrinsic or extrinsic origin: the former represents information coming from specialized receptors within muscles, joints, tendons, and skin and from visual, auditory, and vestibular receptors during and after the production of the required movement [8]. On the contrary, in extrinsic feedback it is the information about an external source that provides an activity to the subject [8]. In any case, the physiological assumption of neurorehabilitation, both motor and cognitive, is represented by the phenomenon of neuroplasticity and the consequent processes of learning and motor control [9,10]. By neuroplasticity, we mean the brain’s ability to modify its structure and function according to its neurons’ activities, which are correlated, for example, to somatosensory stimuli [9].

### 1.2. Traditional Clinical Approaches

In recent years, clinical research has allowed the development of rehabilitation methods that offer innovative therapeutic possibilities to stroke patients.

Two main approaches characterize the methods: a Sherringtonian one (Bobath methods, Kabat, etc.), in which the motor activity is the result of motor commands operated by reflex pathways, and a cortical one (Perfetti’s method), which supports the essential role of the reprogramming of the movement elaborated at the cortical level [11]. According to the Sherringtonian method, there is a temporal relationship of immediacy between stimulus and motor response; on the other hand, according to the school of cortical thought, the exercise proposed to the patient must reach a state of consciousness. The Bobath method bases its principles on inhibiting abnormal reflex activity and facilitating regular postural and motor activity [11,12].

The Perfetti method, on the other hand, proposes therapeutic exercise as a cognitive problem. The use of cognitive processes is therefore recalled, in particular using attention and memory. Furthermore, this model takes into account the body as a receptor surface. With this method, the subject does not learn the movement action again, but its sensations (tactile, pressure, or kinesthetic) [8]. In addition to these techniques, which have now become traditional, researchers have developed innovative rehabilitation approaches in recent years, which often take on a very practical-operational significance.

None of these techniques is an alternative to the others. An effective physiotherapy method is Constraint-Induced Movement Therapy (CIMT), which limits the healthy side’s use and subjects the paretic upper limb to an intensive, repetitive, and functional gesture-oriented exercise [13]. This technique bases its rationale on reestablishing interhemispheric balance, decreasing the somatosensory inputs from the healthy upper limb, simultaneously increasing the information to the affected hemisphere. However, only a few centers can provide this type of therapy as the protocols prescribe a daily rehabilitation treatment for two consecutive weeks and the healthy limb’s overall restriction for at least 90% of the day [13]. Ref. [14] describes the discovery of “mirror neurons” in the monkey’s cortical area activated by observing and performing a movement. This discovery paves the way for a new rehabilitation approach to recover the upper limb’s motor skills. Mirror therapy is a rehabilitation method that involves the movement of both upper limbs in a balanced way; according to this therapy’s protocol, the patient has to observe the healthy limb’s action in the mirror. The patient has the impression that the paretic limb is also moving simultaneously. In this way, the afferents are attributed to the paretic hand and reactivate the circuits in charge [14].

More recently, robotic rehabilitation would allow the intensive administration of somatosensory inputs to the paretic hemisome by repeating motor gestures. Concerning the upper limbs, motor rehabilitation carried out with wearable robots’ aid has proven to be particularly useful if carried out in a very early post-stroke phase [15,16].

### 1.3. Paper Contributions and Organization

In this paper, exploiting recent developments in Robotics-Assisted Therapy (RAT), we present a novel way of giving exergames for post-stroke rehabilitation using a special device consisting of a passive robotic arm. The configurable exergames take advantage of the imitation of daily life actions by proposing the execution of simple tasks. The proposed system aims to become a method used to make patients learn simple movements again and restore higher personal independence levels. To this end, the system embeds a data analysis module with a twofold goal: first, it permits the analysis of the single performance in completing a task; second, it allows the extraction of more general indices for gaining a deeper insight into the neurological post-stroke status. The system has been tested in the lab as well as in a relevant scenario by conducting a preliminary experiment with five patients.

The paper is organized as follows. In Section 2 we recall the state of the art, surveying the main approaches in post-stroke rehabilitation, also considering new emergent robotic technologies. Then, in Section 3, we describe the overall system, detailing the passive robotic arm Track-Hold (Section 3.1), a simple software module for trajectory following (Section 3.2), and, finally, the implemented task-oriented exergames (Section 3.3), focusing on the software features for creating and editing personalized protocols and tasks. In Section 4, the data analysis module is presented first by introducing indices of post-stroke neurological damage and Parkinson-type disorders and, then, by describing a procedure for determining the Range of Movements (ROM) in patients. Finally, Section 5 ends the paper with directions for future development and experimentation.

## 2. State of the Art

### 2.1. Impact of e-Health Solutions and Exergaming Development in Clinical Monitoring and Rehabilitation Practice

In the last few years, several experimental e-health services and Internet of Things (IoT) platforms have been set up, not only in the post-stroke domain but also for the long-term care of frail older people, to prevent and monitor cognitive and physical decline [17,18,19].

While such innovative Information and Communication Technologies (ICT) solutions require facing new issues such as data privacy management [20] in IoT networks, their introduction is justified by the existence of clinical and experimental evidence in their favor. Indeed, advanced applications that make use of virtual reality exergaming and telerehabilitation systems, in addition to traditional therapy, may be effective for functional recovery in post-stroke patients, and also suitable for re-establishing motor skills and balance control in subjects suffering from neurodegenerative disorders, such as Parkinson’s disease [21,22].

On the other hand, concerning severe neurological disorders, such as the most chronic hereditary ataxias, for which there is no current disease-modifying pharmacological therapy, it is assumed that video games, exergames, and serious games are effective as traditional rehabilitation, improving motor outcomes and quality of life [23].

Although other authors report that exergames, both commercial and serious games, improve mobility and balance in healthy older adults with physiological impairment of performances, further investigations are needed to evaluate the real impact of this training on quality of life and activities of daily living [24].

### 2.2. Robotic Assisted Therapy in Post-Stroke Recovery

Clinical practice and epidemiological data suggest that RAT, as a traditional or advanced rehabilitation treatment (i.e., transcranial direct current stimulation), is more effective for motor function recovery in the early stages of post-stroke syndrome, namely the subacute phase, than in chronic convalescence [25,26]. In the chronic stage, functional recovery is slower and involves specific mechanisms of motor learning [27]. In the last two decades, RAT has been applied to motor and cognitive rehabilitation with a different type of technology and executive protocol, but clinical reports show that EMG-driven rehabilitation robots have better outcomes than passive rehabilitation robots [28].

Despite RAT allowing the recovery process of impaired functions, more controlled and randomized clinical trials are required. A recent randomized trial [29] showed that neurocognitive robotic-assisted training of the hand improves motor ability, and is non-inferior compared to traditional dose-matched neurocognitive therapy. Furthermore, in a controlled pilot study, ref. [30] highlighted that patients that enrolled in a robotics-assisted rehabilitation group and trained over 10 days made relevant improvements in the rehabilitation tasks.

Concerning different types of robotics rehabilitation performed in subacute and chronic stage patients with severe-moderate upper limbs paralysis, results of a controlled randomized single-blinded study indicate that end-effector robot intervention is better than exoskeleton robot training. Particularly, there were significant differences between these different robotics interventions regarding the level of activity and participation of patients in the chronic stage [31].

Several relevant studies have suggested that the use of electroencephalography-based brain–computer interfaces can promote the post-stroke restoring process, although, particularly for hand rehabilitation, in the literature systems at prototype or preclinical stages of development are reported [32]. According to the clinical picture’s severity, the therapy carried out with robotic devices involves different protocols (passive, active, or active-assisted movements) [16,33]. No verification exists regarding to what extent the performance improvement extension, required in the robotic context, transfers to daily life activities in the medium to long term. In this regard, it would be useful to continue producing scenarios in the rehabilitation setting that are increasingly similar to those of everyday life so that the required movement can be traced back to targeted action and learned again by the patient. The Track-Task software proposed in this paper was designed and developed following this approach.

## 3. System Architecture

### 3.1. Track-Hold Device

**Figure 1:**
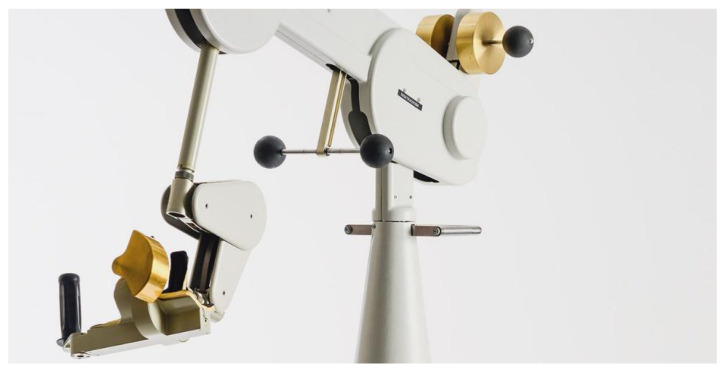
The body of the passive robotic arm called Track-Hold.

Track-Hold is a rehabilitation device for passive training of the upper limbs with gravity support in spatial movements (Figure 1). New position sensing technologies ensure full arm motion detection. Weight relief through gravity compensation facilitates movement in patients with hemiparesis and efficiently permits upper limb rehabilitation (http://www.wearable-robotics.com/kinetek/products/track-hold/, accessed on 20 April 2021). The therapist can easily adjust the amount of support by removing/adding physical weights. This process uses a patented counterbalance mechanism.

**Figure 2:**
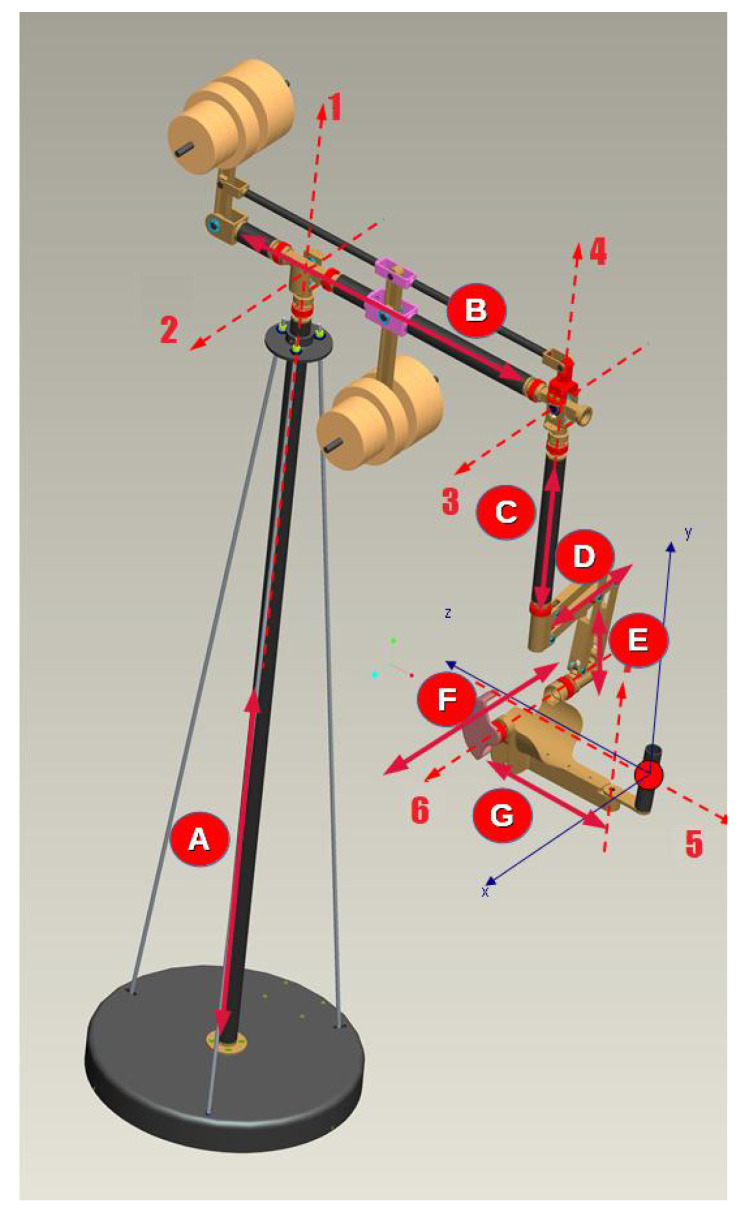
Track-Hold axes. The length of arm A (identified by a number inside a red circle) is about 2200 mm, arm B is about 1200 mm, arm C is about 1000 mm, arm D is about 250 mm, arm E is about 250 mm, arm F is about 250 mm, and G is about 250 mm.

Track-Hold allows to record kinematic data during therapy sessions and, among many applications, estimate daily improvements and progress in the therapy program. Seven rotation sensors enable monitoring the device’s mobile joints’ movements, having an accuracy of about one-tenth of a degree (Figure 2). The device’s manipulator, consisting of a handle, is equipped with a pressure sensor (useful, for example, for detecting the gesture of picking up objects). The sampling rate of the positions is 100 Hz, which is largely sufficient for correct motion detection. The device is connected to a computer using Universal Serial Bus (USB).

### 3.2. Trajectory Software

Trajectory is an exergame designed and developed by Wearable Robotics that simulates the gesture of drawing and erasing a picture drawn on a blackboard. The 3D visualization of the blackboard, the chalk, and the unreal game engine executes the eraser implementation. The Track Hold hand-effector controls the cursor on the whiteboard, represented by a blue ball. The game has two modes: in the first one, the subject must draw a path following a thin trace on the blackboard and then erase it with the eraser. In the second mode, the drawing process is automatic: the subject must follow the path while automatically drawing and erasing it using the eraser. The possible paths are simple shapes such as a circle, a square or an 8-shaped path. At the end of the session, in both modes, Trajectory saves a file containing the exercise data. The exercise data consist of the path drawn on the screen by the subject within the reference trace (the minimal route to execute the exercise). At the end of each session, Trajectory stores the movement path and shows it later in a separate window. The analysis of the route takes place through the functions made available by Matlab and its toolboxes. The use of Trajectory in conjunction with the Matlab environment allows one to experiment and fine-tune the basic mechanisms of Track-Task.

### 3.3. Track-Task Software

With the aim of extending the exergame variety and increasing the therapeutic potential of the Track-Hold device, an additional application, named Track-Task, has been designed and implemented for the execution of neuromotor exercises. Unlike Trajectory, Track-Task exercises follow a functional basis principle. By completing the activities proposed by Track-Task, the subjects regain movements useful for everyday life tasks.

**Figure 3:**
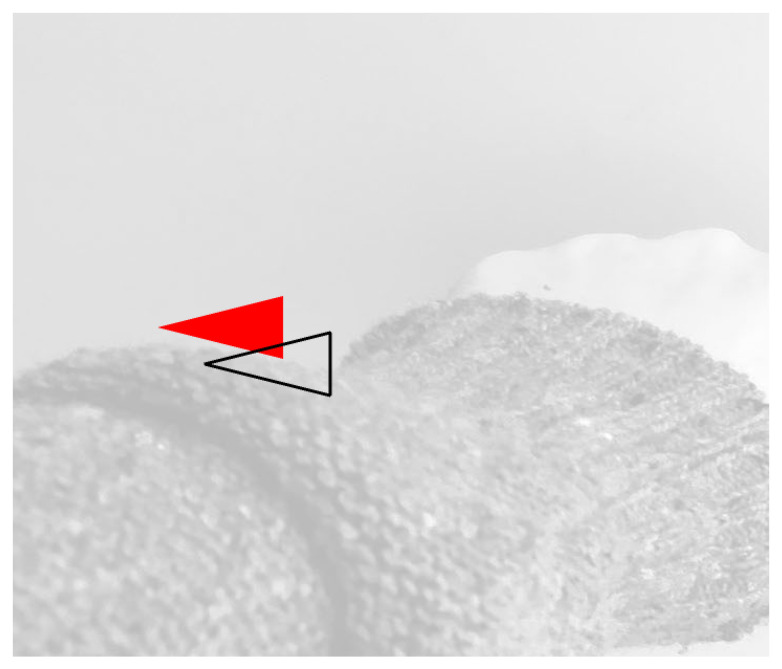
The key-point marked with red and the cursor drawn using black lines.

In detail, the functional movements, performed by manipulating the device, are broken down into elementary sub-movements, which are accomplished when a cursor on the screen reach es key-points, characterized by an exact angle and position. The motion of the manipulator on the X, Y plane is in immediate correspondence with the screen’s position. The variation of the cursor’s size identifies the Z-axis movement: pushing the manipulator forward (Z coordinate increase) decreases its size. This metaphor is intuitive and allows, by diversifying the target key-points’ size and consequently their position on the Z-axis, to guide the subject to make movements throughout the three-dimensional space (Figure 3).

The application works with a dual monitor configuration: the service monitor facing the operator and a large one, intended for viewing the exergame, placed in front of the subject. The application consists of three distinct modules:

- Track-Task Control Panel: an operator on the service monitor manages the Track-Task Control Panel using a Graphical User Interface (GUI). The GUI permits to choose the protocol of the session and to control the execution;
- Track-Task Visualizer: the exergame viewer, intended for full-screen viewing on the monitor in front of the subject;
- Track-Task Editor: a task editor that allows creating new exercises by adding them to the existing ones.

A suitable classification divides the subjects into categories, and there exists a correspondence between categories and protocols. Each protocol specifies a Track-Task session consisting of a set of specific tasks. By following the protocol, the subject must perform a functional value movement due to its characteristics, as it recalls a precise gesture corresponding to daily action.

As already explained, each of the tasks is composed of segmented sub-movements, in which the subject must reach oriented key-points placed in 3D space, moving the manipulator while observing the cursor on the screen. At the moment, a predefined list of tasks available by default mimicks simple daily activities (see Table 1). Notice that using the Track-Task Editor, it is possible to add new custom tasks. Indeed, the editor has a straightforward interface based on mouse and keyboard, through which it is possible to control the insertion of new key-points in the path.

**Figure 4:**
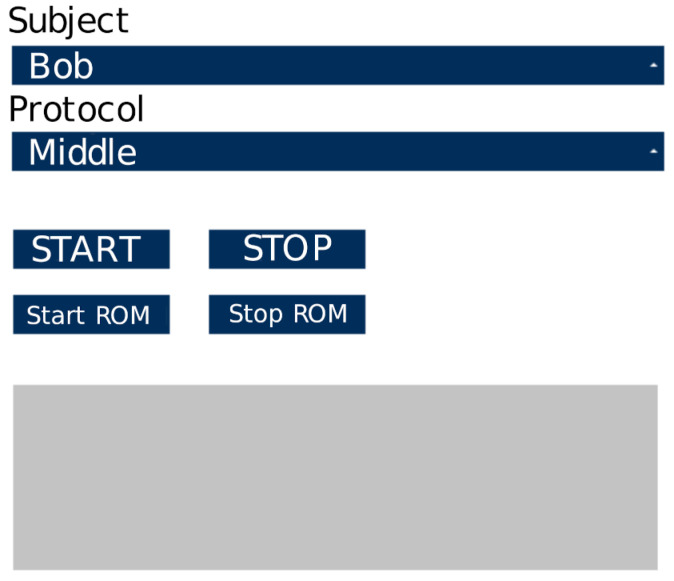
Track-Task control panel.

The Track-Task control panel (Figure 4) deserves a brief overview of the functionalities it offers. Indeed, through the Track-Task control panel it is possible to perform the following actions:

- Selection of subject: a drop-down menu allows the choice of the subject who has to perform the session. A text file contains the list of persons, and it is editable with any editor.
- Protocol choice: a drop-down menu allows one to choose the session protocol. An editable text file contains available protocols. Each of the protocols comprises a list of individual exercises (tasks). The individual tasks are stored in an appropriate folder of the application and can be created and edited using the Track-Task editor.
- Start/Stop ROM: these buttons allow the operator to start or stop the ROM recording. This mode requires the subject to make the movements (the widest he/she can perform) under the operator’s guidance. At the end of the operation (controlled by the Stop ROM button), the software stores the subject’s current range of movements in the data folder.
- Start/Stop: once the subject and protocols are chosen, these buttons allow the medical specialist to start or stop the exergame session related to a subject and a protocol.

## 4. Data Analysis Methodology

This section shows the method starting from the data acquisition methodology to data analysis.

It is interesting evidence that the system can be easily applied to the treatment of most patients. In fact, most patients tolerate this approach.

Preliminary experimentation with five patients started after an initial technical validation phase inside our laboratories. The purpose of this preliminary experimentation was only to technically validate the system in a real scenario. Our pilot project involved a group of five patients in the post-stroke chronic stage. They formed a group of two women (mean age 78.6 ± 4.5 yo) and three men (mean age 75.7 ± 3.2 yo). The patients had partial paralysis of the right upper limb.

### 4.1. Data Acquisition and Analysis

**Figure 5:**
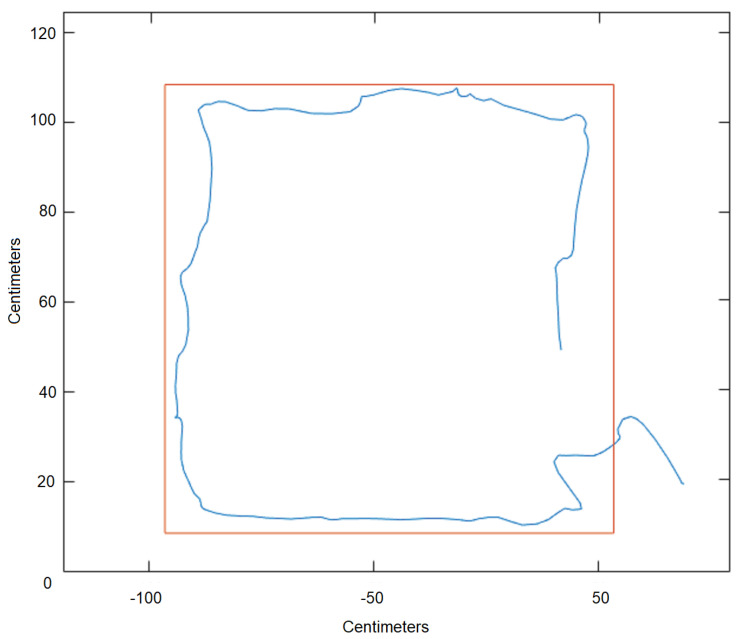
The graph shows the 2D track view on a test square task. The blue line is the path that the patient had to follow, the red line is the executed path by the patient. Track on a test square Task (2D view).

During the exergame sessions, Track-Task acquires all the data relative to the subject’s movements that are crucial to evaluate performance and identify trends. A simple square-shaped template is shown in (Figure 5) in a 2D projection, together with the path drawn by a subject while trying to follow it.

To this end, the data analysis module starts automatically at the end of each task, evaluating a number of features by running *ad hoc* algorithms.

After the computation, the control panel and the primary monitor display the following results:

- *Total time:* total time spent on the task, expressed in seconds;
- *Distance overhead:* a percentage that indicates the actual distance traveled by the cursor in space, compared to the shortest path’s length between the key-points;
- *LF index & HF index:* features deriving from an analysis of the data in the frequency domain.

Among the computed features, a special role is given to the analysis of motion in the frequency domain. Indeed, such an analysis can detect indications of tremors associated with neuromotor disorders as a function of their frequency. In particular, thanks to Track-Task, it is not only possible to record the trajectory followed by the end-effector of the arm and evaluate the precision in the execution of a task, but it is also possible to analyze features of the data more deeply related to neurological conditions. Indeed, several studies conducted with wearable and contact-less sensors [34,35,36], have demonstrated that Low-Frequency (LF) tremors (5–8 Hz) are symptoms of neuro-motor problems, both those related to post-stroke neurological damage and those arising from Parkinsonian-type disorders. High-Frequency (HF) tremors (8–15 Hz) can be classified as systemic or physiological, the extent of which may be a function of the subject’s age.

The frequency characterizes its typology. Without going into detail here, we can say that LF tremors (5–8 Hz) are symptoms of neuro-motor problems, both those related to post-stroke neurological damage and those related to Parkinsonian-type disorders. HF tremors (8–15 Hz) can be classified as systemic or physiological, the extent of which may be a function of the subject’s age. The Track-Task application then performs a frequency analysis of the movement traces to distinguish these two bands. More in detail, the following steps describe the various phases of the data analysis: On the basis of such well-grounded considerations, in Track-Task a data analysis process has been designed and implemented for evaluating LF and HF motion indices. The process is based on the following pipeline taking as input a path, expressed as an array of coordinates, and producing as output the evaluation of two numerical indices.

**Figure 6:**
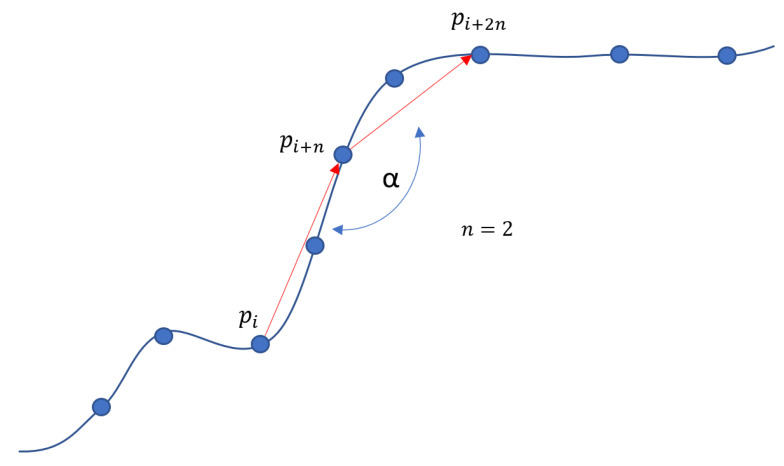
The method used to calculate the one-dimensional array of angles from the series of points in space. 3D path to angle sequence.

1. Transformation of the array of coordinates <x,y,z> into an array of angles <α>: in practice, after having selected a step *n* of the order of some units, the path is scanned progressively, choosing three points $p_{i}$, $p_{i+n}$, $p_{i+2n}$ at each time (Figure 6). However, not being periodic, these angular variations will not be particularly present in the frequency analysis.
2. Fourier transform by Discrete Fourier Transform (DFT). We have chosen to use the canonical implementation instead of the Fast Fourier Transform (FFT) as it does not require that the vector’s length be a power of 2. In our case, the path’s size can be of arbitrary length, and therefore it would have been necessary to resort to techniques such as zero paddings or windowing (Equation (1)):
  (1) $$A_{k}=\sum_{j=0}^{N-1}\alpha_{j}\cdot \left[\cos\left(\frac{2\cdot \pi }{N}\cdot k\cdot j\right)-i\;\cdot \sin\left(\frac{2\cdot \pi }{N}\cdot k\cdot j\right)\right]$$
  where *N* is the length of the array $<\alpha >=<\alpha_{0},\ldots ,\alpha_{j},\ldots ,\alpha_{N-1}>$. Furthermore, in our case, the frequencies to be extracted are limited (we are not interested in the entire obtainable spectrum, from 0 to 50 Hz) and therefore it is possible, by limiting the DFT algorithm only to this part of the range, to reduce by a lot of computation time, to be able to provide analysis results immediately at the end of each task.
3. Band filtering and index calculation: the frequency range extracted varies from 2 to 20 Hz. Within this range, we consider two sub-ranges, LF, between 3 and 8 Hz, and HF from 8 to 15 Hz. The analysis algorithm extracts two simple indices, i.e., (a) the *LF Index* computed as the sum of the modules of the LF band elements (Equation (2)) and (b) the *HF Index* computed as the sum of the modules of the HF band elements (Equation (3)):
  (2) $$LF=\sum_{k=l_{1}}^{l_{2}}\left|A_{k}\right|$$
  (3) $$HF=\sum_{k=h_{1}}^{h_{2}}\left|A_{k}\right|$$

**Figure 7:**
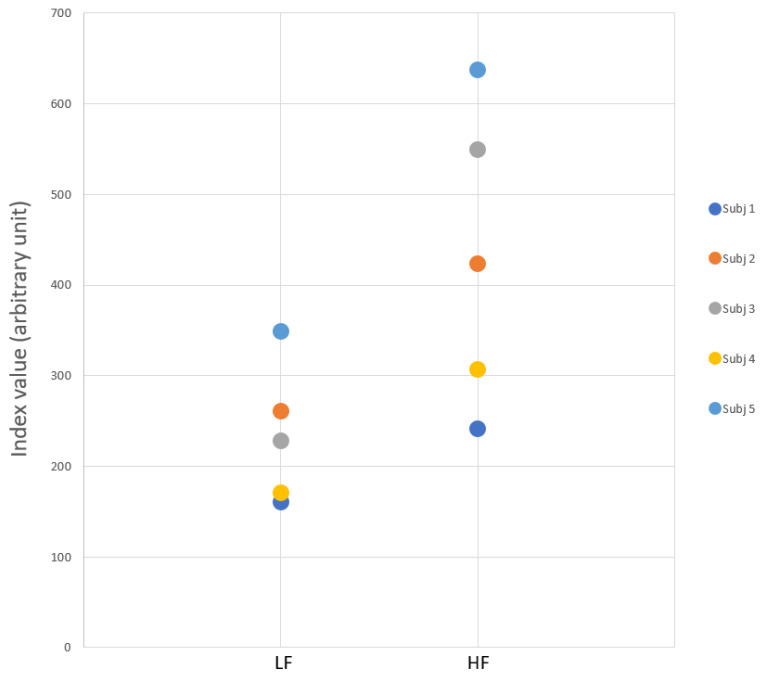
A typical dispersion of the average values of the LF/HF indexes for the 5 subjects, on task 1.

Notice that the values of the two indices are dependent on the type of task, and therefore their evaluation acquires significance only based on the comparison of a normative database built with control subjects. Typical possible values on Task 1 are shown in (Figure 7).

Such a mathematical procedure for the computation of the indices has been integrated into Track-Task in an *ad hoc* interface. In particular, after a task is terminated, the computation is triggered and the control panel and the primary monitor display the following results:

- *Total time:* total time spent on the task, expressed in seconds;
- *Distance overhead:* a percentage that indicates the actual distance traveled by the cursor in space, compared to the shortest path’s length between the key-points;
- *LF index & HF index:* Features deriving from an analysis of the data in the frequency domain.

### 4.2. ROM Measurement

**Figure 8:**
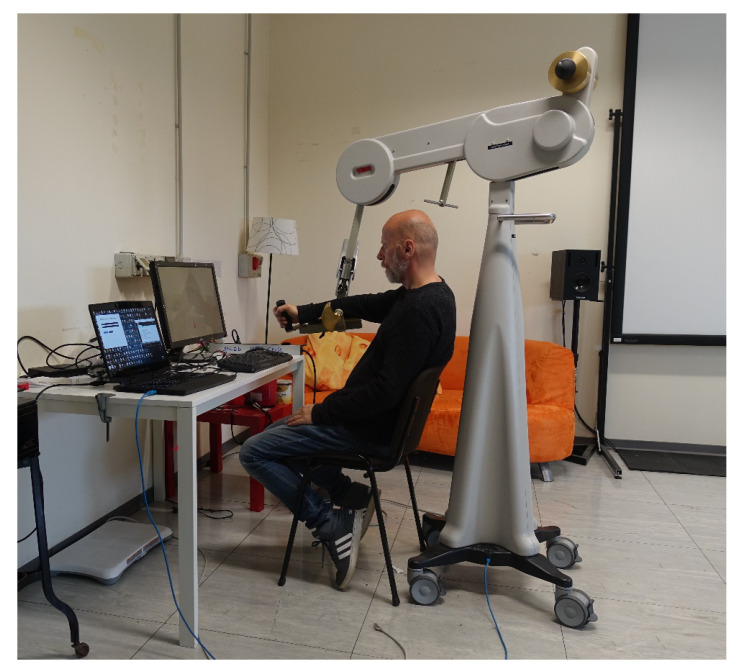
Side view of the system, with operator monitor (laptop) and larger display monitor for the subject.

**Figure 9:**
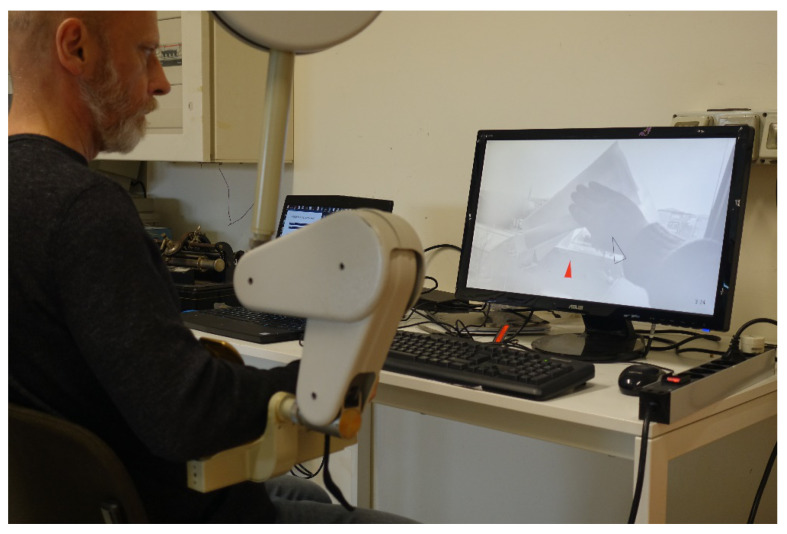
Rear view of the system, with red key-point and 3D cursor displayed on the subject’s monitor.

**Figure 10:**
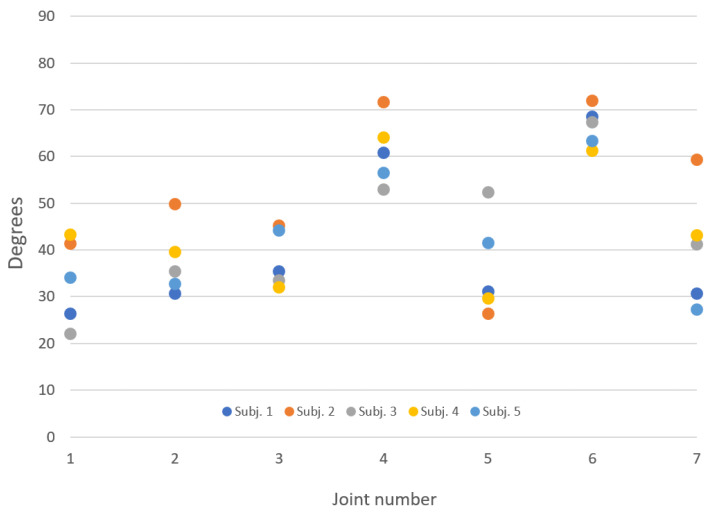
The figure shows for each subject (represented by the colored balls) a typical maximum angular excursion of the seven joints (numbered as in Figure 2). Subject’s, in degrees, for each of the 7 joints.

The software provides a ROM mode, which allows measuring the subject’s maximum movement capabilities. Note that the devices’ constructive features do not make the ROM of the subject’s joints easily measurable: this would require the applications of sensors directly on the subject’s limbs, an operation not compatible with the typical scenario of the use of the system (Figure 8 and Figure 9). Therefore, in practice, the maximum excursion is measured at seven angles of the device’s joints, activated by the subject’s movement (Figure 10). This measurement becomes relevant once a normative database built on controls (healthy subjects) has been created, against which to make a comparison.

## 5. Conclusions

This paper has presented a new category of exergames for post-stroke rehabilitation based on functional task-oriented exercises that mimic movements usually performed in daily life. The ultimate goal is to make the results of robotic rehabilitation therapies more persistent and better centered on everyday tasks in such a way as to establish a virtuous circle for rehabilitation in which movements are traced back and learned again by the patient. To this end, we have introduced the Track-Task software, which, in combination with a passive robotic arm named Track-Hold, represents a complete end-to-end system for rehabilitation. Indeed, the software provides the simulation of scenarios of common day-to-day life task. Besides pre-defined tasks, a task editor allows the creation of new unlimited exercises to meet the subjects’ needs and inclinations better. More importantly, a data analysis module is provided, computing both metrics on the single performance as well as global indices for the evaluation of motion artifacts related to post-stroke neurological damage and Parkinsonian-type disorders. The present robotics rehabilitation system was particularly designed to assist patients in the chronic stage of post-stroke syndrome, performing repetitive movements with their hemiparetic limbs to regain motion and ability in the activities of daily living. Furthermore, this robot does not require a large workspace and high effort for patients that, for the first time, experience machine interaction.

Other features of Track Hold, including a system of gravity compensation, could allow a gradual and progressive recovery of upper limb muscle strength and mobility.

Although further data must be acquired, during the experimental period described in this paper, we observed satisfactory levels of compliance and adherence of patients, a good level of system usability, and, finally, a growing interest of clinicians to assess, as much as possible, personalized training protocols. The system is now about to be validated on a larger group of subjects suffering from post-stroke neuro-motor deficits at the “Tabarracci” Assisted Living Facility (ALF) in Viareggio, Italy. Before this experiment, we plan to carry out a series of trials with control subjects to build the normative database. At the end of the ALF experiment, the envisaged and desirable results are *an increase in ROM capacity, an increase in precision and speed of movements, and a decrease in pathological tremors*.

Among the future developments foreseen, we could imagine some hardware modifications, which facilitate the switch between manipulation with right and left limbs as well as general ergonomics, and some improvements of the software, which can, for example, include an online review system of the recorded data without having to resort to external tools.

## Figures and Tables

**Table 1:** List if available tasks.

Task	Description	Keypoints
Pushing a door	A gesture of pushing a door	2
Switch off	Turning off a switch	2
Opening a right door	A gesture of opening a door opening to the right	3
Opening a left door	A gesture of opening a door opening to the left	3
Open wardrobe	Open a wardrobe with a sliding door	3
Drinking and eating	Bring a bottle/food to the mouth	3
Stir	A gesture of mixing a dish with a ladle	4
Clean a glass	A gesture of cleaning with a cloth	7

## Abbreviations

The following abbreviations are used in this manuscript:

**ADL**: Activity Daily Living
**ALF**: Assisted Living Facility
**CIMT**: Constraint-Induced Movement Therapy
**DFT**: Discrete Fourier Transform
**EMG**: ElectroMyoGraphy
**FFT**: Fast Fourier Transform
**GUI**: Graphical User Interface
**HF**: High-Frequency
**ICT**: Information and Communication Technologies
**IoT**: Internet of Things
**LF**: Low-Frequency
**RAT**: Robotics-Assisted Therapy
**ROM**: Range of Movements
**USB**: Universal Serial Bus
**WHO**: World Health Organization
